# Development and validation of a risk prediction model for overall survival in patients with nasopharyngeal carcinoma: a prospective cohort study in China

**DOI:** 10.1186/s12935-022-02776-8

**Published:** 2022-11-19

**Authors:** Siwei Miao, Haike Lei, Xiaosheng Li, Wei Zhou, Guixue Wang, Anlong Sun, Ying Wang, Yongzhong Wu

**Affiliations:** 1 Department of Health Statistics, School of Public Health, Chongqing Medical University, Chongqing, 400016 China; 2 Chongqing Cancer Multi-Omics Big Data Application Engineering Research Center, Chongqing University Cancer Hospital, Chongqing, 400030 China; 3 MOE Key Lab for Biorheological Science and Technology, State and Local Joint Engineering Laboratory for Vascular Implants, College of Bioengineering, Chongqing University, Chongqing, 400030 China

**Keywords:** Nasopharyngeal carcinoma, Nomogram, Overall survival, Prognosis

## Abstract

**Objective:**

Nasopharyngeal carcinoma (NPC) is prevailing in Southern China, characterized by distinct geographical distribution. Aimed to predict the overall survival (OS) of patients with nasopharyngeal carcinoma, this study developed and validated nomograms considering demographic variables, hematological biomarkers, and oncogenic pathogens in China.

**Methods:**

The clinicopathological and follow-up data of the nasopharyngeal carcinoma patients obtained from a prospective longitudinal cohort study in the Chongqing University Cancer Hospital between Jan 1, 2017 and Dec 31, 2019 ($\mathrm{n}=1635$). Cox regression model was used to tested the significance of all available variables as prognostic factors of OS. And independent prognostic factors were identified based on multivariable analysis to model nomogram. Concordance index (C-index), area under the receiver operating characteristic (AUC), calibration curve, and decision curve analysis (DCA) were measured to assess the model performance of nomogram.

**Results:**

Data was randomly divided into a training cohort (1227 observers, about 70% of data) and a validation group (408 observers, about 30% of data). At multivariable analysis, the following were independent predictors of OS in NPC patients and entered into the nomogram: age (hazard ratio [HR]: 1.03), stage (stage IV vs. stage I–II, HR: 4.54), radiotherapy (Yes vs. No, HR: 0.43), EBV ($\ge 1000$ vs.$<1000$, HR: 1.92), LAR ($>6.15$ vs.$\le 6.15$, HR: 2.05), NLR ($>4.84$ vs. $\le 4.84$ HR: 1.54), and PLR ($>206.33$ vs.$\le 206.33$, HR: 1.79). The C-indexes for training cohort at 1-, 3- and 5-year were 0.73, 0.83, 0.80, respectively, in the validation cohort, the C-indexes were 0.74 (95% CI 0.63–0.86), 0.80 (95% CI 0.73–0.87), and 0.77 (95% CI 0.67–0.86), respectively. The calibration curve demonstrated that favorable agreement between the predictions of the nomograms and the actual observations in the training and validation cohorts. In addition, the decision curve analysis proved that the nomogram model had the highest overall net benefit.

**Conclusion:**

A new prognostic model to predict OS of patients with NPC was developed. This can offer clinicians treatment making and patient counseling. Furthermore, the nomogram was deployed into a website server for use.

## Introduction

Nasopharyngeal carcinoma (NPC) is an epithelial carcinoma originating from the nasopharyngeal mucosal tissue, with the characteristic of distinct geographical distribution of occurrence [1, 2]. NPC occurred highly in East and Southeast Asia [1], and it particularly prevalent in Guangdong and Guangxi, the regions of southern China [3]. In 2019 the number of NPC deaths in China reached 28,659, accounting for 40% of NPC deaths worldwide [4]. China accounts for a significant proportion of mortality of NPC over the world, especially in southern China [5, 6].

Epstein-Barr virus (EBV) is one of the most common causative agents, and it can be detected in all types of NPC [7]. Radiotherapy is the primary treatment choice for NPC treatment due to the radiosensitive characteristic of NPC tumor [8]. And precise staging is crucial for reducing mortality in patients with NPC. However, heterogeneities of clinical outcomes of the NPC patients with the same clinical stage and degree of EBV were reported in considerable recent research. Those findings indicated that it is not enough to refine the prediction of outcomes for NPC patients only considering single factors. Recently, a major current focus in the area of prognostic of NPC is to find more risk factors to get a more accurate predictive model. Numerous studies have demonstrated that hematological biomarkers were associated with survival outcomes of NPC patients, such as lymphocyte-albumin ratio (LAR), neutrophil–lymphocyte ratio (NLR), platelet-to-lymphocyte ratio (PLR) [9–11]. However, the literature related to the survival outcomes of the cohort among NPC patients that take various factors into account in model design are limited. Therefore, in this study, our objective was to develop a clinically useful prognostic model in which demographic variables, hematological biomarkers, and oncogenic pathogens were considered to predict overall survival among NPC patients in the region.

## Materials and methods

### Data source

The data set used in the retrospective cohort study was obtained collecting 1635 patients with NPC from the Chongqing University Cancer Hospital tumor database between Jan 1, 2017 and Dec 12, 2019. The inclusion criteria were as follows: (1) age $\ge 18$ years; (2) histologically confirmed primary NPC; (3) the main treatment occurred in our hospital; (4) completed baseline clinical information and follow-up information; (5) completed the entire course of the treatment of radiotherapy, chemotherapy and targeted therapy. The exclusion criteria for this study were as follows: no follow-up records and a history of cancer treatments. The present study was performed according to the guidelines of the Declaration of Helsinki and was approved by the ethics committee of the Chongqing University Cancer Hospital. Written informed consent was obtained from all subjects.

### Variables

IN this study, we employed demographics, including age, sex (female and male), ethnicity (Han and others), marriage (married and others), and occupation (worker/clerk, self-employed/unemployed, professional and technical personnel, and others). The clinical characteristics were selected, including clinical stage which was classified according to the American Joint Committee on Cancer Staging Manual (8th edition), pathological (non-keratinized differentiation, non-keratinized undifferentiated, and keratinized squamous cell carcinoma and others), and transfer information. We also abstracted therapeutic methods information like radiotherapy, chemical-therapy and targeted-therapy. Finally, we retrieved laboratory variables, which consisted of EBV, LAR, NLR, and PLR and selected the cutoff point using X-tile. Continuous variables in laboratory data were transformed into categorical variables based on cutoff values: EBV ($<1000$, $\ge 1000$), LAR ($\le 0.13$, $>0.13$), NLR ($\le 4.84$,$>4.84$), PLR ($\le 206.33$, $>206.33$).

The endpoint of interest in this study was the overall survival (OS) of NPC patients, calculated from the date of first diagnosed as NPC to the time of death or the last follow-up was set as the end.

### Construction of nomogram

Patients were randomly divided into train group (1227 observers, about 70% of data) and a validation group (408 observers, about 30% of data). The nomogram model was developed using training cohort. The univariate Cox regression analysis was performed to verify the prognostic significance of each covariate as factor of OS. And entered the variables with p-value $<0.05$ to the multivariate Cox regression model to analyze the association between each variable and OS to find independent risk factors. The nomogram was created based on the risk score calculated by the final Cox regression model that was constructed by stepwise process.

### Model performance and validation

Concordance index (C-index) and area under the receiver operating characteristic (AUC), calibration curve, and decision curve analysis (DCA) were used to assess the model performance of nomogram. C-index was used to estimate the accuracy of the model calculating the difference between predicted value and actual one. The calibration curve was evaluated using a plot to estimate the performance of accordance of the prediction and e reality. DCA calculates a clinical “net benefit” for one or more prediction models in comparison to default strategies of treating all or no patients.

### Statistical analysis

The data analysis was performed using SPSS software version 26.0 (IBM Corp, Armonk, NY) and R software version 4.2.1 (Institute for Statistics and Mathematics, Vienna, Austria) were used. The R packages ‘survival’ (version 3.3-1), ‘foreign’ (version 0.8-82), ‘rms’ (version6.3-1), ‘timeROC’ (version 0.4), ‘rms’ (version 5.0.1) and ‘ggDCA’ (version 5.0.1) were used to develop and evaluate the model. In addition, the R packages ‘rsconnect’ (version 0.8.27) and ‘DynNom’ (version 5.0.1) were employed for developing a webserver of nomogram of NPC. The statistical significance of the two-sided $p$ was set at $\le 0.05$.

## Results

### Characteristics of the training and validation cohorts

Patients with nasopharyngeal carcinoma enrolled in follow-up visit were randomly split between training (n = 1227, 70%) and validation cohorts (n = 408, 30%), from the Chongqing University Cancer Hospital tumor database platform. The median survival time was 27.50 (0.10–135.30) months for the overall cohort. There were 180 deaths over 27.40 (0.10–126.00) months for the training cohort. The validation cohort comprised 69 deaths and the median survival time was 28.00 (0.10–135.30).

The descriptive data of our population are shown in Table 1, and there are no significantly difference in training and testing data set. Overall, the considerable amount of patients were Han (1414, 86.48%), married (1507, 92.17%) male (1189, 72.72), with premetastatic (1493, 91.31%), stage IV (700, 42.81%) and pathological performance status of non-keratinizing differentiation (953, 58.29%). And the mean age of patients was $51.62\pm 11.15$. With regard to therapy, the majority of patients refused targeted therapy (1197, 73.21%). All enrolled cases received chemotherapy for the major choice (772, 62.92%) and 59.09% of patients treated by radiotherapy.

**Table 1:** Patient demographics and clinical characteristics

Characteristics	All patients	Training cohort	Validation cohort	P
	No. (%)	No. (%)	No. (%)	
Total	1635	1227	408	
Gender				0.979
Male	1189 (72.7)	893 (72.8)	296 (72.6)	
Female	446 (27.3)	334 (27.2)	112 (27.5)	
Age (mean (SD))	51.72 (11.0)	51.62 (11.2)	52.027 (10.5)	0.513
Ethnic				0.694
Han	1414 (86.5)	1064 (86.7)	350 (85.8)	
Others	221 (13.5)	163 (13.3)	58 (14.2)	
Marital status				0.464
Married	1507 (92.2)	1127 (91.9)	380 (93.1)	
Others	128 (7.8)	100 (8.2)	28 (6.9)	
Occupation				0.101
Worker/clerk	289 (17.7)	226 (18.4)	63 (15.4)	
Self-employed/unemployed	598 (36.6)	447 (36.4)	151 (37.0)	
Professional and technical Personnel	141 (8.6)	114 (9.3)	27 (6.6)	
Others	607 (37.1)	440 (35.9)	167 (40.9)	
Stage				0.610
I–II	262 (16.0)	203 (16.5)	59 (14.5)	
III	673 (41.2)	502 (40.9)	171 (41.9)	
IV	700 (42.8)	522 (42.5)	178 (43.6)	
Pathological				0.427
Non-keratinizing differentiation	953 (58.3)	720 (58.7)	233 (57.1)	
Non-keratinized undifferentiated	660 (40.4)	493 (40.2)	167 (40.9)	
Keratinized squamous cell carcinoma and others	22 (1.4)	14 (1.1)	8 (2.0)	
Transfer				0.850
No	1493 (91.3)	1119 (91.2)	374 (91.7)	
Yes	142 (8.7)	108 (8.8)	34 (8.3)	
Radiotherapy				0.321
No	681 (41.7)	502 (40.9)	179 (43.9)	
Yes	954 (58.4)	725 (59.1)	229 (56.1)	
Chemical therapy				0.184
No	622 (38.0)	455 (37.1)	167 (40.9)	
Yes	1013 (62.0)	772 (62.9)	241 (59.1)	
Targeted therapy				0.163
No	1197 (73.2)	887 (72.3)	310 (76.0)	
Yes	438 (26.8)	340 (27.7)	98 (24.0)	
EBV				0.659
< 1000	923 (56.5)	697 (56.8)	226 (55.4)	
≥ 1000	712 (43.6)	530 (43.2)	182 (44.6)	
BQ				1.000
≤ 0.13	178 (10.9)	134 (10.9)	44 (10.8)	
> 0.13	1457 (89.1)	1093 (89.1)	364 (89.2)	
LAR				0.521
≤ 6.15	1438 (88.0)	1075 (87.6)	363 (89.0)	
> 6.15	197 (12.1)	152 (12.4)	45 (11.0)	
NLR				0.744
≤ 4.84	1444 (88.3)	1086 (88.5)	358 (87.8)	
> 4.84	191 (11.7)	141 (11.5)	50 (12.3)	
PLR				0.633
≤ 206.33	1290 (78.9)	972 (79.2)	318 (77.9)	
> 206.33	345 (21.1)	255 (20.8)	90 (22.1)	

### Independent prognostic factors in the training cohort

In the training cohort (n = 1227), the independent prognostic factors were performed using Cox proportional hazards models and modeled results were reported in Table 2. The following variables were significant as predictors of OS on univariable analysis: age, occupation (only professional and technical personnel), stage, radio therapy, chemical therapy, EBV, LAR, NLR, and PLR (all $p<0.05$). Reddy etc. found that keratinization may be a vulnerable aid in predicting response to therapy for NPC [12]. And Luo etc. demonstrated that differentiation is close to EBV, which indicates that it was a link between EBV and NPC [13]. Based on clinical consensus and the previous research, we kept the pathological in the model. On multivariable analysis, age (hazard ratio [HR]: 1.03; 95% confidence interval [CI] 1.01–1.04), stage (stage IV vs. stage I–II, HR: 4.59; CI 2.28–9.25), radio therapy (HR: 0.42; CI 0.27–0.66), EBV (HR: 1.98; CI 1.41–2.79), LAR (HR: 2.01; CI 1.41–2.86), NLR (HR: 1.52; CI 1.02–2.28) and PLR (HR: 1.71; CI 1.20–2.44) were demonstrated to be independent predictors.

**Table 2:** Univariate and Multivariate Analysis for overall survival of the Training Cohort

Characteristic	Univariate		Multivariate	
	HR (95% CI)	P	HR (95% CI)	P
Age	1.04 (1.03–1.06)	< 0.001	1.028 (1.014–1.043)	< 0.001
Gender				
Female	1			
Male	1.23 (0.875–1.73)	0.234		
Ethnic				
Han	1			
Others	1.37 (0.94–2)	0.101		
Marital status				
Married	1			
Others	0.845 (0.47–1.52)	0.573		
Occupation				
Worker/clerk	1			
Self-employed/unemployed	0.979 (0.62–1.55)	0.929		
Professional and technical personnel	2.06 (1.23–3.45)	0.006		
Others	1.42 (0.934–2.16)	0.100		
Stage				
I–II	1		1	
III	2.4 (1.18–4.88)	0.016	2.341 (1.145–4.786)	0.020
IV	6.56 (3.33–12.9)	< 0.001	4.593 (2.281–9.248)	< 0.001
Pathological				
Non-keratinizing differentiation	1		1	
Non-keratinized undifferentiated	1.07 (0.794–1.440)	0.663	1.084 (0.804–1.463)	0.597
Keratinized and others	2.27 (0.718–7.150)	0.163	3.130 (0.957–10.236)	0.059
Transfer				
No	1			
Yes	0.97 (0.580–1.620)	0.907		
Radiotherapy				
No	1		1	
Yes	0.368 (0.268–0.505)	< 0.001	0.421 (0.268–0.662)	< 0.001
Chemical therapy				
No	1		1	
Yes	0.469 (0.347–0.634)	< 0.001	0.895 (0.581–1.380)	0.615
Targeted therapy				
No	1			
Yes	0.986 (0.699–1.390)	0.938		
EBV				
< 1000	1		1	
≥ 1000	3.01 (2.22–4.08)	< 0.001	1.982 (1.408–2.792)	< 0.001
BQ				
≤ 0.13	1			
> 0.13	0.907 (0.581–1.420)	0.669		
LAR				
≤ 6.15	1		1	
> 6.15	4.41 (3.22–6.04)	< 0.001	2.009 (1.408–2.712)	< 0.001
NLR				
≤ 4.84	1		1	
> 4.84	2.61 (1.83–3.71)	< 0.001	1.521 (1.015–2.278)	0.042
PLR				
≤ 206.33	1		1	
> 206.33	2.35 (1.73–3.19)	< 0.001	1.712 (1.202–2.438)	0.003

### Developing the prognostic nomogram model

**Fig. 1:**
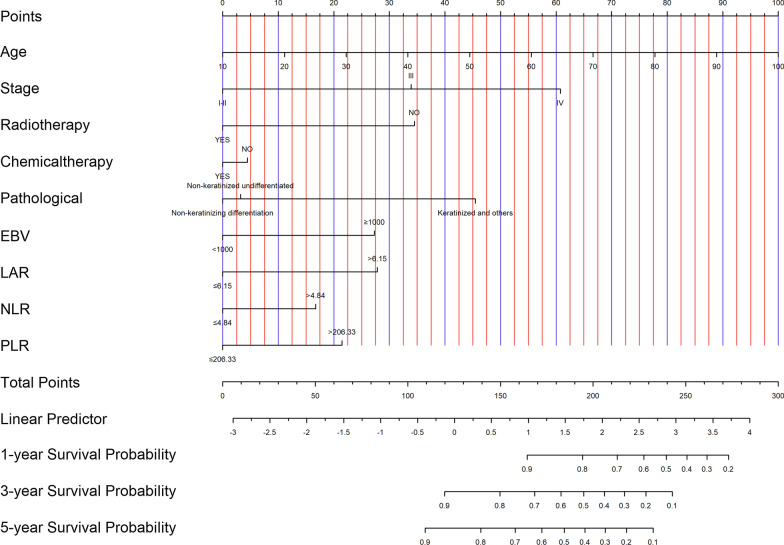
Nomogram model predicting 1-, 3- and 5-year OS in NPC patients in training cohort. The nomogram was used summing the points identified on the points scale for each variable. The total points projected on the bottom scales indicate the probability of 1-, 3- and 5-year survival

Independent predictors on multivariable analysis were selected for the development of nomogram model to predict 1-, 3- and 5-year OS in nasopharyngeal carcinoma patients (Fig. 1). Each variable was converted to a point score based on corresponding Cox estimated regression coefficients and the sum of the values was positioned to the total point table to obtain the probability of OS.

### Model performance and validation of the nomogram

**Fig. 2:**
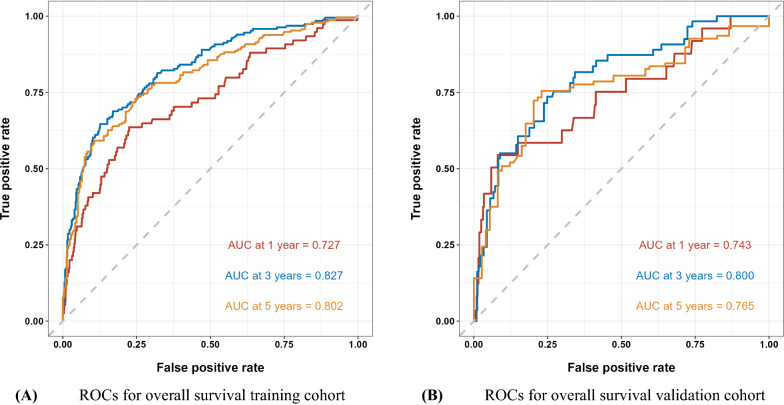
**A** ROCs for overall survival training cohort; **B** ROCs for overall survival validation cohort

**Fig. 3:**
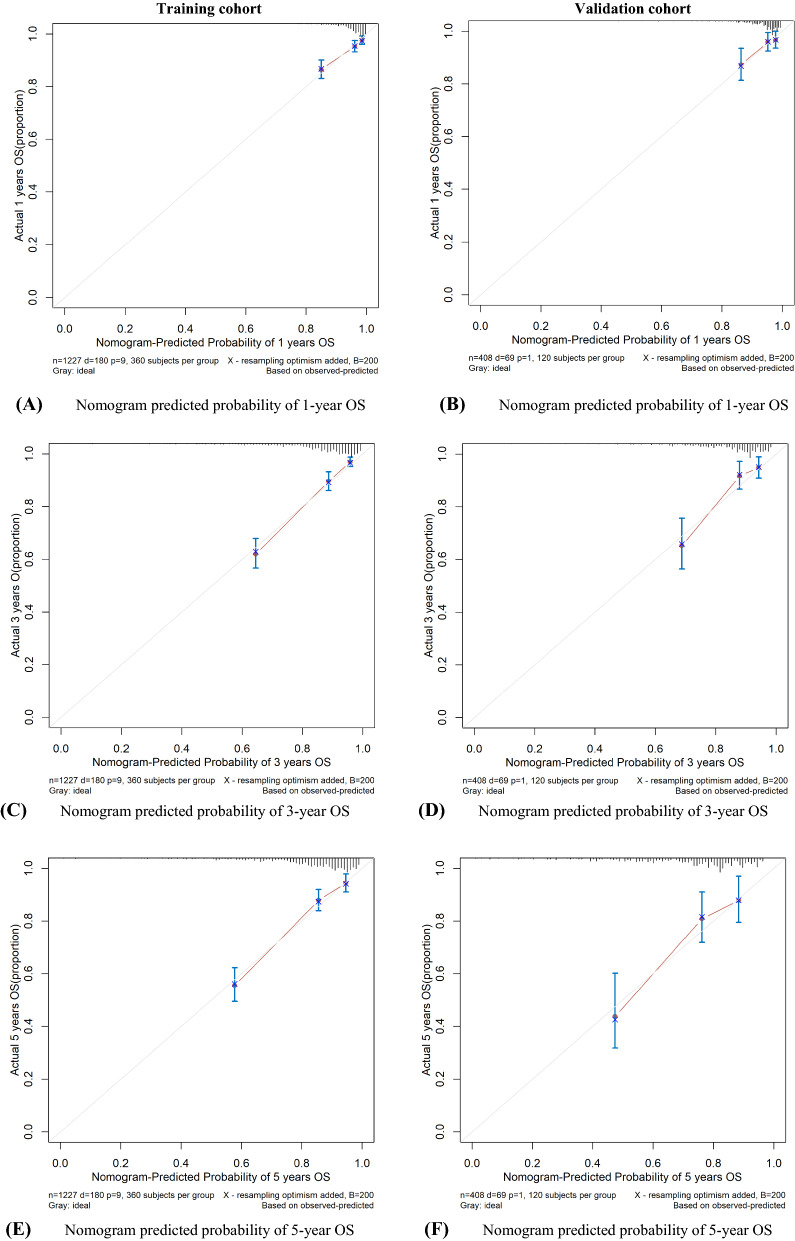
The calibration curves for predicting patient OS at 1,3 and 5 years in the training cohort and at 1,3 and 5 years in the validation cohort. Nomogram model-predicted OS is plotted on the x-axis; actual OS is plotted on the y-axis. Closer alignment with the diagonal with the diagonal line represents a better estimation

**Fig. 4:**
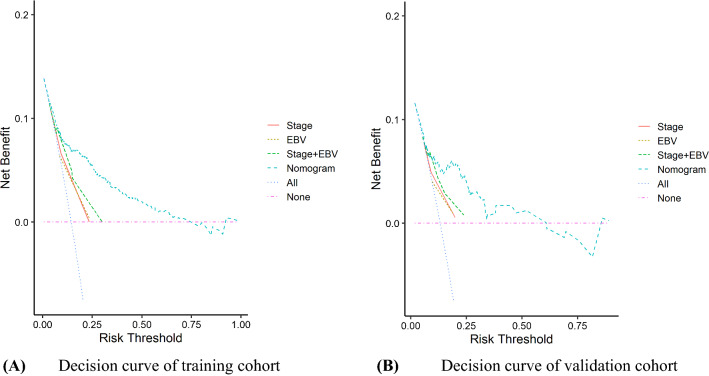
Decision curves analysis for survival predictions

The C-indexes for training cohort at 1-, 3- and 5-year were 0.73(95% confidence interval [CI] 0.66–0.79), 0.83 (95% CI 0.79–0.86), 0.80 (95% CI 0.75–0.85), respectively. In the validation cohort, the C-indexes was 0.74 (95% CI 0.63–0.86), 0.80 (95% CI 0.73–0.87) and 0.77 (95% CI 0.67–0.86), respectively. And ROC plots presented in Fig. 2. In addition, the calibration curve at 1-, 3-, 5-year survival of the model performed well, showing good agreement between the predictions of the nomograms and the actual observations in the training and validation cohorts (Fig. 3.). Moreover, the decision curve analysis was used to test the predictive ability of the nomograms. The DCA results of the four models showed that, except for a small range of predicted probability threshold between 75 and 90%, the nomogram model displayed a positive net benefit in the train set (Fig. 4.).

### Risk-stratifying ability of the nomogram

**Fig. 5:**
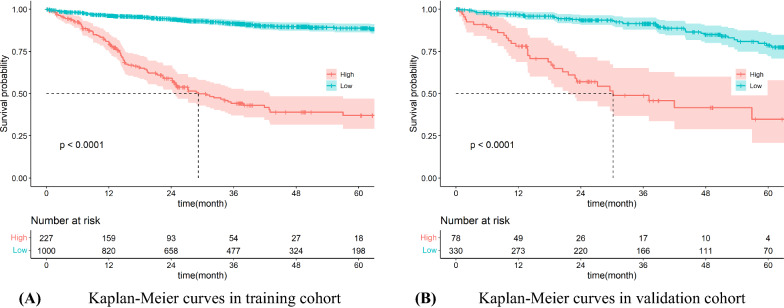
Kaplan–Meier curves of the nomogram stratification methods Determinations of risk score groups based on the predictive risk scores for overall survival in the overall in the training and validation cohorts

Based on the predictive risk scores calculated by the nomogram model, the study subcategorized the training and validation cohort into low-risk group (the prognostic risk score was less than the threshold) and high-risk group (the prognostic risk score was greater than the threshold). And the Kaplan–Meier survival curves for OS presented significant differences between the two groups in the training and validation cohort ($p<0.0001$) (Fig. 5).

### Webserver development for the nomogram

**Fig. 6:**
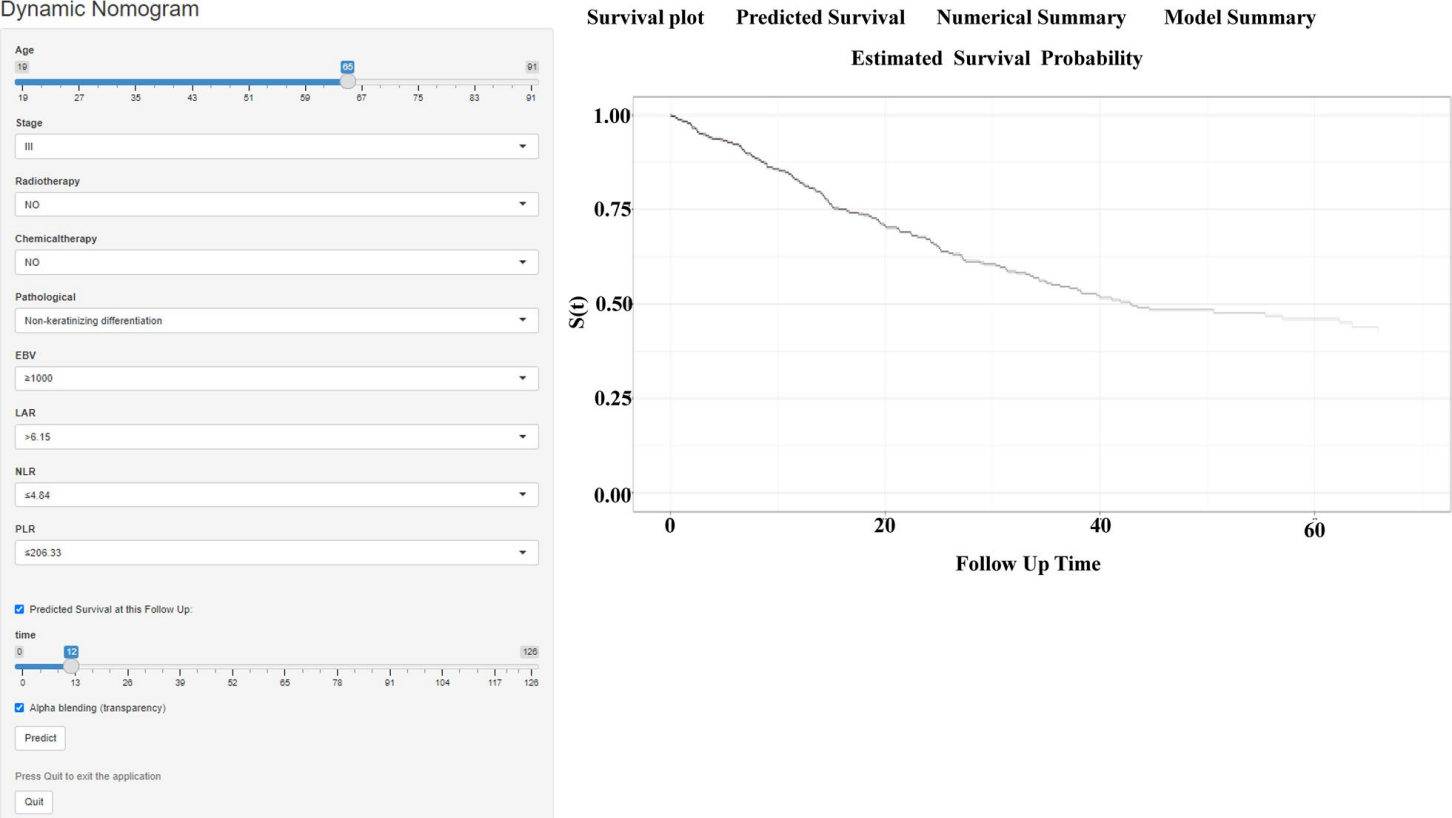
The interface of the web-based nomogram

We developed an easily accessible webserver for the nomogram model of NPC (https://nomogramwebserverofnpc.shinyapps.io/DynNomapp/). The survival plot and probability of the patient can be displayed by selecting the corresponding indexes and survival time on the left side of the webserver board (Fig. 6). For example, the probability of one patient with the following characteristics at 1-year is 0.82: 65-year-old, stage 3, with non-keratinizing differentiation, no radiotherapy, no chemical-therapy, EBV ≥ 1000, LAR > 6.15, NLR ≤ 4.84, PLR ≤ 206.33, and the probability of the patient with same characteristics at 3-year and 5-year was 0.55, 0.46, respectively.

## Discussion

In the present study, we used the follow-up database from the Chongqing University Cancer Hospital to establish a novel nomogram prognostic model of NPC and complete internal verification, by incorporating demographics, hematological biomarkers, and oncogenic pathogens. And a user-friendly online calculator was developed to help clinicians in treatment decision making.

Several previous studies have been published using the nomogram to predict the OS of NPC patients. In 2018, Wu and colleagues evaluated a nomogram for predicting long-term OS for patients wish NPC using demographic variables and TNM stage [14]. And Huang etc. developed a prognostic nomogram to reveal the relationship between EBV and NPC in 2021 [15]. Although the western region is not a high incidence area of nasopharyngeal carcinoma, the survival prediction of its patients should not be ignored. Inflammatory markers have been widely used in various cancers, but rarely in NPC, thus we established a prognostic model considered several systemic inflammation parameters and EBV.

Several of our findings are worth highlighting. First, age, stage, radio therapy, EBV, LAR, NLR and PLR were recognized as independent prognostic parameters based on the univariate and multivariate cox regression analysis, and the conclusion was in general agreement with previous reports [9, 16–18]. EBV infection is the most common causal agent [19] and a useful prognostic factor of NPC [20], and has been used to assess the disease progression and population screening [21]. LAR is a novel independent prognostic risk factor [9] and have a strong survival predictive power for OS in NPC [18]. Li etc. concluded that NLR could be an attractive indicator for evaluating the 5-year OS in NPC patients with stage III [22]. High PLR was associated with poor OS in NPC patients [23]. Notably, chemical therapy was suggested that it did not reach statistical significance to be a prognostic factor in our study, and it was unlike some previous results [24]. Considering the chemotherapy sensitizing the tumor to the toxic effects of the radiotherapy [25] and the choice to chemotherapy depending on clinical risk, for example, the results obtained in the study is reasonable. Radiotherapy is the primary curative treatment of NPC, and combing chemotherapy with radiotherapy is a rational option in the treatment of locoregionally advanced NPC [26]. Therefore, to avoid missing important factors and based on the clinical features, we conduct model incorporating chemotherapy. In addition, it should be noted that through the univariate model, the correlation between pathological and NPC was of no significance, which was contradictory to other researches.

In our study, the calibration curve pointed optimal accordance between predicted survival probability and actual value, which indicated good repeatability and reliability of the model. And the C-index presented the same performance of our model, in the range of 0.72–0.82 (in training and validation cohorts). In addition, the DCA curves illustrated a better performance of survival predictions of nomogram than the models with stage, EBV, and stage + EBV. In conclusion, the results were suggested that our nomogram was a reliable and precise prognostic tool to predict OS in NPC patients.

Our study is not devoid of limitations. First, there may exist a potential source for selection bias based on the serious inclusion and exclusion criteria. Second, our samples were collected from a single center from a non-endemic region in China and lack of external verification. It is necessary for our study, aimed at exploring the performance of combination of OS and disease-free survival, to design a multicenter randomized controlled study in the next step.

## Conclusion

Patients with NPC have heterogeneous survival outcomes, which can be predicted using our novel prognostic model. And it can support help in clinicians deciding treatment and patient counseling. Furthermore, the nomogram was deployed into a website server for use.

## Data Availability

The raw data supporting the conclusion of this article will be made available by the authors, without undue reservation, to any qualified researcher.
